# An mRNA Vaccine Encoding Rabies Virus Glycoprotein Induces Protection against Lethal Infection in Mice and Correlates of Protection in Adult and Newborn Pigs

**DOI:** 10.1371/journal.pntd.0004746

**Published:** 2016-06-23

**Authors:** Margit Schnee, Annette B. Vogel, Daniel Voss, Benjamin Petsch, Patrick Baumhof, Thomas Kramps, Lothar Stitz

**Affiliations:** 1 CureVac AG, Tübingen, Germany; 2 Friedrich-Loeffler-Institut, Greifswald-Insel Riems, Germany; Wistar Institute, UNITED STATES

## Abstract

Rabies is a zoonotic infectious disease of the central nervous system (CNS). In unvaccinated or untreated subjects, rabies virus infection causes severe neurological symptoms and is invariably fatal. Despite the long-standing existence of effective vaccines, vaccine availability remains insufficient, with high numbers of fatal infections mostly in developing countries. Nucleic acid based vaccines have proven convincingly as a new technology for the fast development of vaccines against newly emerging pathogens, diseases where no vaccine exists or for replacing already existing vaccines. We used an optimized non-replicating rabies virus glycoprotein (RABV-G) encoding messenger RNA (mRNA) to induce potent neutralizing antibodies (VN titers) in mice and domestic pigs. Functional antibody titers were followed in mice for up to one year and titers remained stable for the entire observation period in all dose groups. T cell analysis revealed the induction of both, specific CD4+ as well as CD8+ T cells by RABV-G mRNA, with the induced CD4+ T cells being higher than those induced by a licensed vaccine. Notably, RABV-G mRNA vaccinated mice were protected against lethal intracerebral challenge infection. Inhibition of viral replication by vaccination was verified by qRT-PCR. Furthermore, we demonstrate that CD4+ T cells are crucial for the generation of neutralizing antibodies. In domestic pigs we were able to induce VN titers that correlate with protection in adult and newborn pigs. This study demonstrates the feasibility of a non-replicating mRNA rabies vaccine in small and large animals and highlights the promises of mRNA vaccines for the prevention of infectious diseases.

## Author Summary

Although first successful vaccination against rabies virus infection was performed by Louis Pasteur in the 19^th^ century, every year about 50,000 patients, predominantly children, succumb to rabies infection because of insufficient availability of effective low-cost vaccines worldwide. The work presented here describes the protective capacity of such a vaccine candidate based on a non-replicating messenger RNA (mRNA). Here we highlight the efficacy of this type of vaccine in a highly fatal viral infection mouse model and demonstrate the induction of accepted correlates of protection in domestic pigs. The results extend and strengthen our previous work on mRNA-based vaccines protecting against Influenza. The data from Rabies and Influenza studies, together with the increased thermostability (manuscript in preparation) and the conceived cost-effectiveness of production suggest that non-replicating mRNA-based vaccines are an attractive and promising format for the development of protective vaccines against a wide range of infectious diseases.

## Introduction

Rabies is an invariably fatal neurological disease that affects different species of warm-blooded animals, including wild animals, pets, and humans. This infectious disease is caused by a strictly neurotropic virus. The rabies virus has a bullet-shaped, enveloped virion with a negative-sense single-stranded RNA genome that encodes five viral proteins: nucleoprotein, phosphoprotein, matrix protein, glycoprotein, and RNA-dependent RNA polymerase. Human rabies cases are almost exclusively caused by animal bites, in particular by dogs. After the incubation phase, humans first develop a flu-like illness and thereafter severe neurotropic symptoms caused by the ensuing progressive encephalomyelitis. While incubation phases vary, death commonly follows within an average survival time between 6 and 11 days after first symptomatic onset for furious or paralytic forms, respectively, thus leaving little time and extremely limited therapeutic options [1,2].

The virus also replicates in salivary glands of infected dogs and is thus commonly transmitted through bite wounds, licking of damaged skin, or direct mucosal contact. Enhanced aggressiveness of rabid animals results in an effective transmission strategy. The virus attaches to its cellular targets by the surface glycoprotein (RABV-G), rapidly gains access to peripheral nerves, and then, after retrograde axonal transport and trans-synaptic spread, ultimately reaches the brain. Transport of the enveloped virus within nerve cells and neuronal transport vesicles impedes clearance by humoral or cellular immunity [3–6].

As a consequence, effective immunological defense against rabies must intercept virus before productive neuronal infection. This may require immediate neutralization by antibodies directed against the viral G protein upon entry of rabies virus into uninfected tissue and/or early elimination of infected cells by virus-specific cytotoxic T cells, when limited replication may take place in non-nervous tissue at the site of entry. This is most effective when the neutralizing antibodies and clearing T cells are already present *in situ* at the time of infection, which is the case after prophylactic vaccination. In contrast thereto, protection has first to be built up in case of initiation of vaccination after exposure to the rabies virus, which requires time, as it is done by PEP vaccination. That is the reason for the administration of rabies immunoglobulin (RIG) together with active PEP vaccination in the case of suspected rabies exposure in rabies naïve individuals. Rabies vaccines were introduced historically by Louis Pasteur and have been used for more than a century, mostly to control canine rabies that poses the highest risk for transmission to humans [7]. While vaccinating animals has greatly reduced human cases, transmission via stray dogs or other species, such as bats and raccoons, remains problematic in many countries [8,9]. Despite the availability of effective vaccines, rabies infection continues to claim at least 55,000 human lives per year (WHO Weekly epidemiological record, No. 49/50, 2007), not counting the great excess of fatal infections in animals. In India, more human fatalities are caused by rabies than in any other country of the world with an estimated 16,000 deaths in the year 2010 alone [10]. Thus, the global provision of safe, effective, and affordable vaccines for use in humans remains important. A special challenge is the adequate supply of vaccine doses to impoverished persons in resource-poor settings. This medical need warrants continued development of alternative vaccine technologies to further reduce the prohibitive cost of currently available egg-based or cell-culture derived vaccines [11], to prevent shortage of vaccine supply [12], and to facilitate distribution.

Early rabies vaccines were produced in mammalian neural tissue and have been replaced by vaccines manufactured in tissue culture and embryonated eggs over time. Novel vaccine formats such as subunit, DNA or viral vector vaccines have been assessed successfully for their protective capacity against rabies infection in preclinical settings [3,13] but none of these resulted in a licensed product for human use yet.

In this study, we assessed the immunogenicity and protective capacity of rabies-specific mRNA vaccines. Messenger RNA (mRNA) vaccines are a genetic vaccine format that may address short-comings of current vaccine technologies. Benefits are the induction of balanced and enduring immunity as demonstrated for anti-tumor and prophylactic vaccination [14–16], simple supply, and storage at elevated temperature, as reviewed elsewhere [17,18]. In addition, it is common view that production for mRNA vaccines is cost-effective [19–21]. The mRNA used here as a vaccine formulation (RNActive) includes optimization of coding and non-coding elements of the mRNA molecule, its purification after *in vitro* transcription and its formulation with protamine for enhanced adjuvanticity [15,17,22–24].

In mice, intradermally injected mRNA encoding RABV-G induced humoral and cellular immunity with a clear dose-response relationship. The format conferred full protection in a stringent murine challenge model of intracerebral inoculation with rabies virus. We show that protection is dependent on the presence of CD4+ T cells during immunization. Moreover, we demonstrate the early, efficient viral clearance and brain homeostasis by mRNA vaccination upon intracerebral infection. Finally, immunogenicity was shown in domestic pigs representing a relevant animal model with a skin highly similar in its architecture to the human skin [25,26]. Here virus neutralizing antibody responses were induced that are well above the WHO threshold of 0.5 IU/ml in adult as well as in newborn pigs.

In the course of the clinical testing of this new substance class, the expert advisory panel of the WHO assigned the suffix “-MERAN” as international nonproprietary name (INN) to mRNA drug substances, and nadorameran to the drug substance of the mRNA rabies vaccine [27].

## Materials and Methods

### Virus

Rabies virus CVS-11 was grown on BHK-21 cells and used throughout the experiments.

### mRNA- and protein-based vaccines

All mRNA vaccines were based on the RNActive platform (EP1857122 and WO2012019780A1). mRNA vectors contained a 5’ cap structure, 5’ UTR, open reading frame (ORF), 3’ UTR, polyA tail and did not include chemically modified nucleotides. In brief, optimization entailed GC-enrichment of the open reading frame (EP 1392341 and EP 1800697), inclusion of enhanced UTRs, and complexation with protamine (Valeant Pharmaceuticals, Eschborn, Germany) as described elsewhere [14,15]. The mRNA rabies vaccine encodes the glycoprotein (RABV-G) of the Pasteur strain (GenBank accession number: AAA47218.1). Two different optimized mRNA constructs were used for immunization (RABV-G A and B) containing identical ORFs but different UTRs. Except for the analysis of the longevity of the T cell response, mice were immunized with RABV-G mRNA A. Pigs where immunized with RABV-G mRNA B. In all immunization experiments, mRNA was complexed with protamine. Both mRNA sequences were product candidates and therefore both used for preclinical tests. The full sequence for both mRNA constructs is given in S1 Fig. The mRNA was produced by T7-polymerase-based *in vitro* run-off transcription [28,29]. The RABV-G mRNA vaccine encoded the full-length, structurally unaltered rabies virus glycoprotein. The licensed vaccines Rabipur (Novartis) and HDC (human diploid cell vaccine) are commercially available and were purchased from a local pharmacy. Either Rabipur or HDC were used as positive controls dependent on the availability.

### Mice and pigs

Mice (BALB/c and C57BL/6, 6–8 weeks of age) were obtained from Janvier Laboratories (Le Genest-Saint-Isle, France), Charles River Laboratory (Sulzfeld, Germany) or the animal breeding facilities at the Friedrich-Loeffler-Institute, Tübingen, Germany. Female pregnant pigs (*Sus scrofa domesticus*) and adult Hungarian large whites were purchased from local breeders. Studies with adult pigs were conducted at Aurigon-Toxicoop Research Center Ltd., Dunakeszi, Hungary. All animal experiments were conducted according to German and Hungarian laws and guidelines for animal protection. Experiments in Germany were approved by the regional council Tübingen (reference numbers FLI238/08, FLI242/09, FLI246/09, CUR 4–13).

### Protein expression

For protein expression analysis, HeLa cells were transfected with RABV-G mRNA or mRNA encoding hemagglutinin (negative control; HA from A/Netherlands/602/2009). 24 h after transfection, cells were stained with a monoclonal mouse anti-rabies antibody (#3R7; HyTest Ltd, Turku, Finland) and a FITC-labelled goat anti-mouse IgG (Life technologies GmbH, Darmstadt, Germany). Expression was detected by analysis of FITC positive cells by flow cytometry.

### Immunizations procedure

Before immunization, mice were anesthetized by i.p. application of ketamine (Sanofi-Aventis, Frankfurt, Germany) and rompun (Bayer, Leverkusen, Germany). Intradermal injection of 100 μl (distributed to two adjacent sites) was performed into the skin in the middle of the back using syringe and forceps. Mice were treated with doses ranging from 1.25–80 μg mRNA. Pigs were immunized intradermally on the upper back with a volume of 100 μl (80μg) or 2x150 μl (240μg) using the “Mantoux” technique. Rabipur and HDC were injected i.m. For mice the i.m. applied injection volume of 100 μl was distributed to four injection sites in the hind limps. Pigs were treated with the full dose of Rabipur (1 ml). For negative control groups, Ringer-Lactate solution or mRNA encoding Ovalbumin was injected.

### Antibody analysis

Blood samples were taken by retro-orbital bleeding (mice) or *vena cava superior* (pigs). Anti-rabies serum antibodies were analyzed by FAVN test by Eurovir Hygiene-Institut, Luckenwalde, Germany according to WHO protocol [30].

### T cell analysis

The induction of antigen-specific T cells was determined using intracellular cytokine staining (ICS) or ELISPOT assay. For ICS splenocytes from vaccinated and control mice were isolated and stimulated with the RABV-G peptide library (JPT Peptide Technologies GmbH, Berlin, Germany) and anti-CD28 antibody (BD Biosciences, Heidelberg, Germany) for 6 hours at 37°C in the presence of the mixture of GolgiPlug/GolgiStop (Protein transport inhibitors containing Brefeldin A and Monensin, respectively; BD Biosciences, Heidelberg, Germany). After stimulation cells were washed and stained for intracellular cytokines using the Cytofix/Cytoperm reagent (BD Biosciences, Heidelberg, Germany) according to the manufacturer’s instructions. The following antibodies were used for staining: CD8-PECy7 (1:200), CD3-FITC (1:200), TNFα-PE (1:100), IFN-γ-APC (1:100) (eBioscience, Frankfurt, Germany), CD4-BD Horizon V450 (1:200) (BD Biosciences, Heidelberg, Germany) and incubated with FcγR-block diluted 1:100. Aqua Dye was used to distinguish live/dead cells (Invitrogen, Life Technologies GmbH, Darmstadt, Germany). Cells were collected using a Canto II flow cytometer (Beckton Dickinson, Heidelberg, Germany). Flow cytometry data were analyzed using FlowJo software (Tree Star, Inc, Ashland, USA.). For ELISPOT analysis mouse splenocytes were stimulated with 1:20 diluted Rabipur (Novartis) or 0.35 mg/ml bovine serum albumin (Sigma-Aldrich). Secreted IFN-γ was detected using a standard ELISpot protocol and measured using a plate reader (Immunospot Analyzer, CTL Analyzers LLC).

### T cell depletion

BALB/c mice were treated by intraperitoneal injection with monoclonal antibodies (YTS 191 [31]) against the CD4+ T cell population one day before and after the vaccinations. During the first immunization phase, mice were treated with 200 μl (0.3 mg) of 1:50 diluted ascites fluid of YTS 191 antibodies. During the second immunization phase, CD4+ T cells were depleted using a 1:10 dilution. To control efficacy of T cell depletion EDTA-blood probes were stained with fluorochrome conjugated antibodies for 30 min at 4°C using mAbs to mouse CD3 (CD3ε chain), CD4 (L3T4) and CD8a (Ly-2) (BD Bioscience, Heidelberg, Germany) two days after each vaccination. After the incubation time, cells were washed and analyzed by the MACSQuant Analyzer and MACSQuantify software (Miltenyi, Bergisch-Gladbach, Germany).

### Challenge infection

Median lethal virus doses (LD_50_) were determined in BALB/c mice by endpoint titration [32]. Challenge virus was applied by intracerebral (i.c.) injection with a volume of 20 μl and an infectious dose of 40-fold LD_50_. Body weight and clinical signs of challenged mice were assessed daily over a period of two weeks after infection. Mice with less than 75% of initial body weight were sacrificed.

### RNA isolation and quantitative RT-PCR

Mice were sacrificed and perfused to wash out blood cells. Next, brains were harvested and divided into telencephalon and cerebellum. Organs were homogenized using the FastPrep-24 instrument (MP Biomedicals, Eschwege, Germany). Total RNA was isolated from the organ homogenates using TRIZOL reagent (Life Technologies, Darmstadt, Germany). For quantitative RT-PCR, 50 ng RNA was used to determine the expression of different mouse proteins utilizing the iScript one step RT-PCR Kit with SYBR Green (Bio-Rad, Munich, Germany) according to the manufacturer’s protocol and the CFX96 Touch Real-Time PCR Detection System and its software (Bio-Rad). The following specific primers for qRT-PCR were used: Mm_Gapdh_3_SG (NM_008084), Mm_Tnf_1_SG (NM_013693) and Mm_Ifng_1_SG (NM_008337) (Qiagen, Hilden, Germany). The protocol for qRT-PCR started with an incubation at 50°C for 10 min. After this first step, probes were heated to 95°C for 5 min, followed by 40 cycles of 95°C for 10s and 60°C for 30 s and plate reading for green fluorescence. Afterwards, melt curve data were collected from 60°C to 90°C at a ramping rate of 0.2°C per second. Relative expression values to uninfected controls were normalized to the expression value of the housekeeping gene glyceraldehyde-3-phosphate dehydrogenase (GAPDH).

Detection of viral N-protein RNA was performed with the same protocol for qRT-PCR using the following oligonucleotides: 5-GAT CCT GAT GAY GTA TGT TCC TA-3’ (forward), 5’-RGA TTC CGT AGC TRG TCC A-3’ (reverse) [33] purchased at Metabion, Germany.

### Statistics

Statistical analysis was performed using GraphPad Prism software, Version 6.00. Statistical differences between groups were assessed by the Mann Whitney, Kruskal-Wallis, one-way ANOVA Tukey’s multiple comparisons or Dunnett’s multiple comparisons test.

## Results

### Induction of long-lasting virus neutralizing titers in mice

To verify antigen expression, HeLa cells were transfected with mRNA encoding rabies virus glycoprotein (RABV-G) of the Pasteur strain (GenBank accession number: AAA47218.1). Presence of translated glycoprotein at the cell membrane was demonstrated by flow-cytometric cell surface staining using a RABV-G specific antibody indicating protein expression and proper trafficking to the cell membrane. Cells transfected with mRNA encoding influenza hemagglutinin served as negative control (**S2 Fig**).

**Fig 1:**
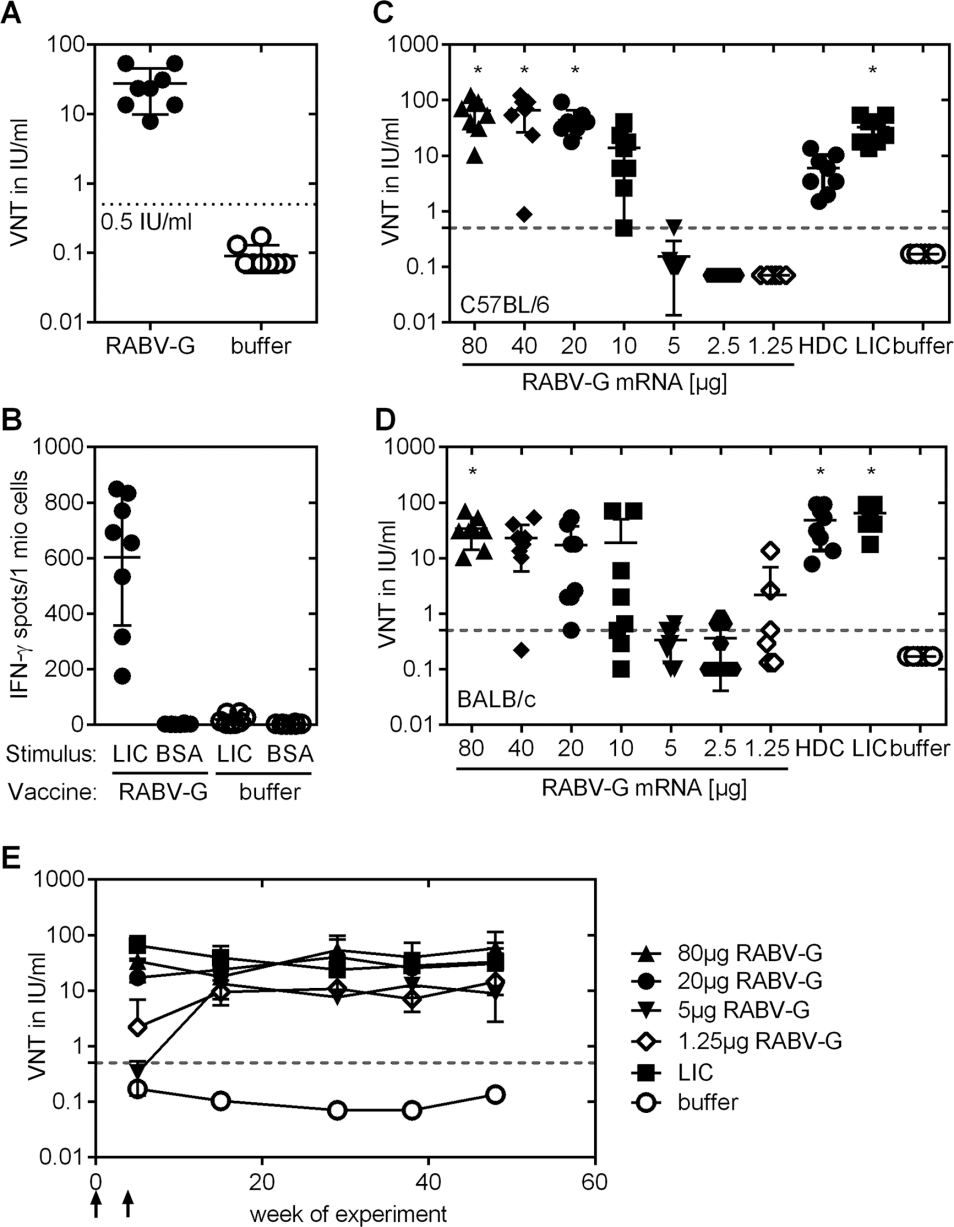
Antigen-specific immune response in mice. ***(A)*** Virus-neutralizing (VN) antibody titers in the serum of female BALB/c mice were measured using Fluorescent Antibody Virus Neutralization (FAVN) test. Animals were vaccinated twice with 80 μg RABV-G mRNA or buffer at day 0 and 21. 35 days after the first vaccination, serum was collected. The dotted line at 0.5 IU/ml is considered as a correlate of protection. ***(B)*** Splenocytes from the same mice were isolated for the analysis of antigen specific T cells via ELISPOT analysis at day 35 after the first immunization. ***(C*, *D)*** VN titers dose-response profile in female ***(C)*** C57BL/6 and ***(D)*** BALB/c mice after RABV-G mRNA vaccination compared to licensed vaccines. Animals were vaccinated with RABV-G mRNA, licensed vaccines (HDC or Rabipur (LIC)) or buffer on study days 0 and 21. RABV-G mRNA was applied intradermally (i.d.) at doses of 80 μg, 40 μg, 20 μg, 10 μg, 5 μg, 2.5 μg or 1.25 μg. For positive control, 100 μl (0.1 human dose) of Rabipur and HDC were administered intramuscularly (i.m.). Presence of rabies-specific VN titers in sera of vaccinated and control mice were analyzed 2 weeks after immunization using the FAVN test. In both graphs the mean and standard deviation (SD) is plotted and significance compared to buffer treated mice was analysed using one-way ANOVA Dunnett’s multiple comparisons test (* p<0.01). ***(E)*** Kinetic of virus-neutralizing antibodies in BALB/c mice from ***(D)*.** Immunization points are indicated by arrows. Mean an SD is presented (n = 8/group).

After showing the expression of functional RABV-G *in vitro*, we next tested whether RABV-G mRNA vaccine was able to induce antigen-specific immune responses *in vivo*. BALB/c mice were immunized on days 0 and 21 with 80 μg of the RABV-G mRNA or buffer. Two weeks after the second immunization, blood was drawn for antibody analysis and splenocytes were isolated for the analysis of antigen specific T cells. After two vaccinations, all RABV-G immunized mice developed relevant virus neutralizing (VN) titers of ≥0.5 IU/ml (range: 5.9 to 70.2 IU/ml), considered as protective in humans, dogs and cats [34]. Buffer treated mice were negative for rabies specific antibodies (**Fig 1A**). RABV-G mRNA vaccine induced antigen-specific T cells were analyzed by ELISPOT assay after stimulation of isolated spleen cells with Rabipur, a licensed rabies vaccine containing inactivated rabies virus. This analysis revealed that in splenocytes of RABV-G mRNA vaccinated mice, IFN-γ secreting cells were detected upon Rabipur stimulation, but not after stimulation with BSA (negative control) or in splenocytes of buffer treated control mice (**Fig 1B**).

We addressed the dose-response relationship of the RABV-G vaccine and evaluated if the observations of the initial experiment in BALB/c were strain specific. BALB/c mice produce predominantly Th2 helper cells and might therefore develop a better antibody response. In contrast, C57BL/6 mice exhibit a Th1 bias and more pronounced cellular immune responses [35]. Female C57BL/6 (**Fig 1C**) and BALB/c mice (**Fig 1D**) were vaccinated on days 0 and 21 with vaccine doses ranging from 1.25 μg to 80 μg mRNA. For positive control, mice received the licensed vaccines HDC or Rabipur. Due to volume restrictions we vaccinated mice intramuscularly with 100 μl (0.1 human doses) of HDC and Rabipur, previously shown to be protective in mice [36,37]. Two weeks after the second immunization, 100% of C57BL/6 and 90% (29 of 32) of BALB/c mice injected with ≥10 μg RABV-G mRNA vaccine developed virus neutralizing (VN) titers of ≥0.5 IU/ml. Thus, in both mouse strains, a dose-response correlation was seen and high neutralizing antibody titers were induced at doses ≥10 μg mRNA. In C57BL/6 mice immunization with a dose of 10 μg RABV-G mRNA vaccine (range: 0.5 to 40.6 IU/ml) revealed comparable immunogenicity to HDC (range: 1.5 to 13.5 IU/ml) and significantly higher titers were achieved at mRNA doses of ≥20 μg (range: 17.8 to 92.4 IU/ml). In BALB/c mice immunogenicity for HDC (range: 7.8 to 92.4 IU/ml) and Rabipur (range: 17.8 to 92.4 IU/ml) equaled that of ≥40 μg RABV-G mRNA (range: 0.2 to 70.2 IU/ml).

These experiments, therefore, indicate comparable immunogenicity of the mRNA vaccine with licensed rabies vaccine and the general induction of specific immune response in mice.

Prophylactic vaccination must also induce durable protection. We tested the longevity of the humoral immune response. BALB/c mice treated with two vaccinations on days 0 and 21 and doses of 1.25 μg, 5 μg, 20 μg, and 80 μg RABV-G mRNA vaccine or 0.1 human dose Rabipur were observed for about one year and antibodies in the serum of vaccinated mice were analyzed monthly. All mice treated with the RABV-G vaccine developed high titers of neutralizing antibodies. We found that after vaccination with 20 μg and 80 μg RABV-G mRNA or with Rabipur antibody titers remained stable throughout the observation period with mean titers between 17 and 65 IU/ml (**Fig 1E**). For the 1.25 μg and 5 μg mRNA vaccine doses, response onset was delayed, but caught up by week 12 of the experiment and titers remained at a high level of ≥ 10 IU/ml. Inter-individual variability was low in all test groups.

### Induction of antigen-specific CD4+ and CD8+ T cells

**Fig 2:**
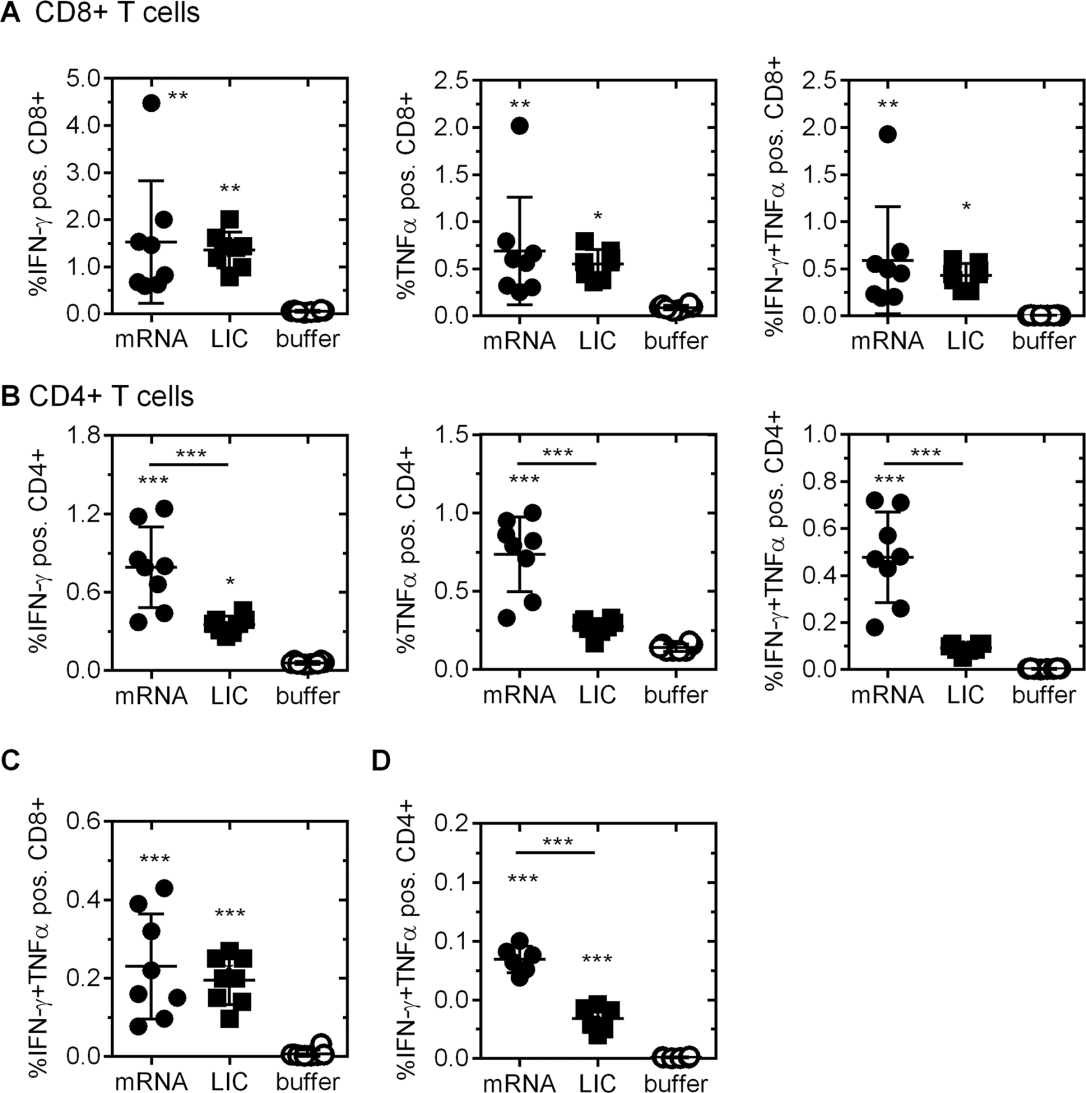
Induction of rabies virus specific T cells upon immunization of female BALB/c mice with RABV-G mRNA. Mice were vaccinated twice (days 0 and 21) with 80 μg RABV-G mRNA, 0.1 human dose Rabipur (LIC) or buffer. Activated, antigen specific ***(A)*** CD8+ T cells and ***(B)*** CD4+ T cells were analyzed by intracellular staining of IFN-γ alone *(left panel)*, TNFα alone (*middle panel*) or IFN-γ and TNFα *(right panel)* followed by flow cytometry analysis. Isolation of splenocytes was done 6 days after the second immunization. ***(C+D)*** Analysis of long-lasting T cell immunoreactivity. Mice were treated with 80 μg RABV-G mRNA, 0.1 human dose Rabipur (LIC) or injection buffer alone at days 0 and 21. Ten weeks after boost, animals were sacrificed and IFN-γ and TNFα double positive antigen-specific ***(C)*** CD8+ and **(*D)*** CD4+ T cells were analysed by flow cytometry (n = 8/group). Mean and SD (n = 8/group) is presented. Statistical significance was tested with one-way ANOVA test using Tukey’s multiple comparisons test compared to buffer group or as indicated (* p≤0.05; ** p≤0.01; *** p≤0.0001).

Although T cells do not prevent initial viral infection of host cells, they provide important impact on rabies virus clearance in mice [38]. Rabies-specific T cell clones have also been isolated from human vaccinees [39]. Thus, we analyzed cellular immune response induced by RABV-G mRNA vaccine in more detail and used the licensed rabies vaccine Rabipur and buffer as positive and negative control group, respectively. We analyzed the presence of antigen specific activated T cells by measuring cytokine induction upon stimulation with a RABV-G spanning overlapping peptide library. Cytokine producing cells were detected by a flow cytometry based method for intracellular cytokine staining (**Fig 2**). Six days after a second vaccination with 80 μg RABV-G mRNA or 0.1 human dose of Rabipur, frequencies of antigen-specific interferon gamma (IFN-γ) and tumor necrosis factor alpha (TNFα) positive CD8+ and CD4+ T cells were detected in all vaccinated mice. The frequencies of RABV-G specific CD8+ T cells of mice receiving Rabipur or the RABV-G mRNA vaccine were comparable (e.g. IFN-γ/TNFα-double positive CD8+ T cells with a mean of 0.59% after RABV-G mRNA and 0.43% after Rabipur vaccination) (**Fig 2A, right panel**). In contrast, antigen-specific CD4+ T cells were significantly elevated in mRNA immunized mice compared to Rabipur treated animals with a high proportion of IFN-γ/TNFα-double positive CD4+ T cells (mean of 0.48% for IFN-γ/TNFα expressing CD4+ T cells after RABV-G mRNA and 0.091% after Rabipur vaccination) (**Fig 2B, right panel**). These results indicate efficient RABV-G-specific T cell induction by RABV-G mRNA and better induction of CD4+ T cells by the mRNA vaccine compared to Rabipur.

In addition, we analyzed the longevity of the cellular immune response induced by RABV-G mRNA. Ten weeks after the second injection (study day 91) splenocytes were isolated from vaccinated and control mice and antigen-specific CD8+ and CD4+ T cells were analyzed upon specific stimulation (**Fig 2C**). In splenocytes of all mice treated with 80 μg RABV-G mRNA or Rabipur, low, but significant frequencies of activated, antigen-specific CD8+ T cells were detected, characterized by staining of intracellular TNFα and IFN-γ (**Fig 2C, left panel**). A significantly higher activity of IFN-γ and TNFα expressing CD4+ T cells was detected after mRNA (mean of 0.084%) compared to Rabipur (mean of 0.034%) immunization (**Fig 2C, right panel**). Frequencies of antigen-specific CD4+ T cells were dose dependent and declined with the dose of RABV-G mRNA used for vaccination (**S3 Fig**).

### Protection against lethal intracerebral challenge in mice

**Fig 3:**
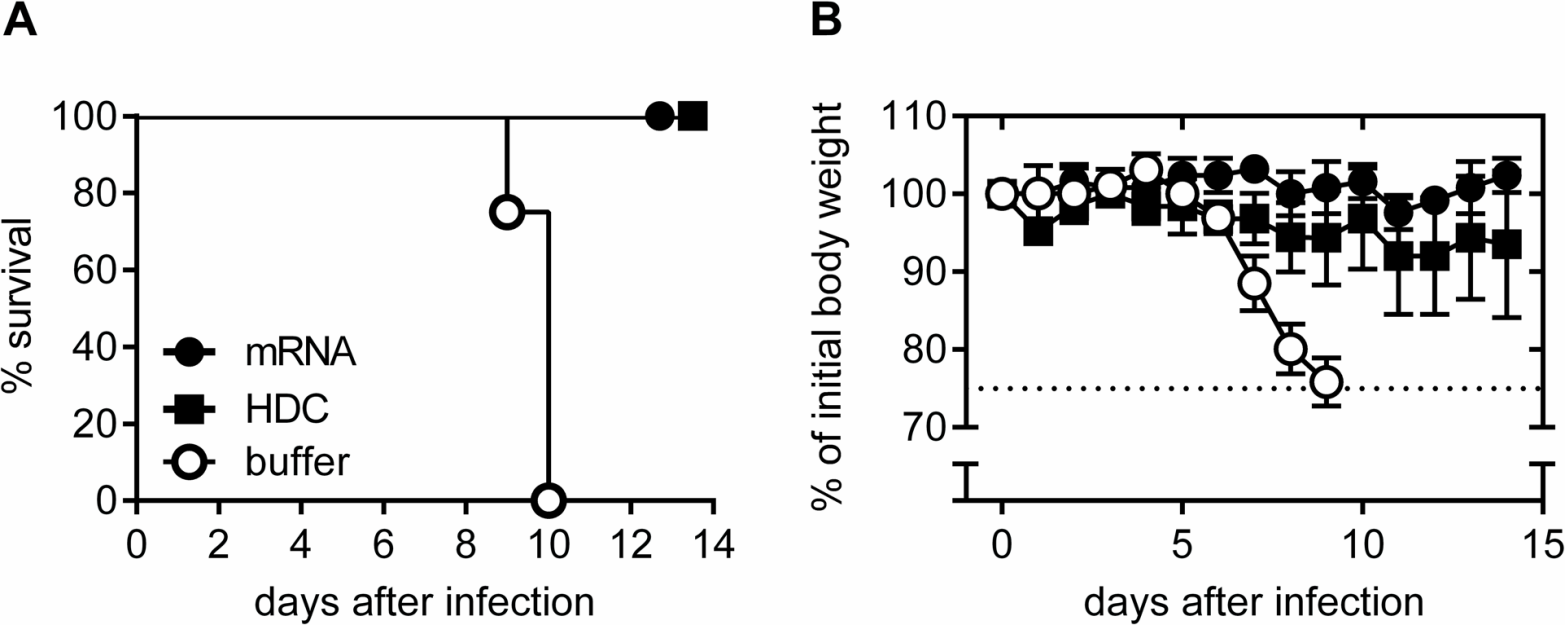
Protective capacity of mRNA vaccine against lethal intracerebal (i.c.) rabies challenge infection. Female BALB/c mice were vaccinated twice (days 0 and 21) with 80 μg RABV-G mRNA, 0.1 human dose HDC or injection buffer alone as negative control. Six weeks after the second immunization, mice were infected i.c. with rabies virus. ***(A)*** Survival of challenged mice is illustrated by a Kaplan-Meyer-analysis. ***(B)*** Body weight of challenged mice was monitored daily. As soon as animals lost 25% of initial body weight (dotted line), animals were sacrificed for reasons of humane endpoint criteria (n = 5 /group). Mean and SD is presented.

To test the protective capacity of the RABV-G mRNA vaccine against rabies infection, RABV-G mRNA vaccinated mice were challenged intracerebrally (*i*.*c*.) with infectious rabies virus of strain CVS-11 (challenge virus standard-11). Intracerebral infection poses an extremely harsh challenge that is artificial, but accepted by the scientific community as a stringent experimental test. Importantly, it also forms the basis of the widely used National Institutes of Health (NIH) potency test for rabies vaccines [40]. BALB/c mice were vaccinated on days 0 and 21 with 80 μg RABV-G mRNA. Positive control mice were vaccinated intramuscularly with 0.1 human dose of HDC, negative control mice received injection buffer (Ringer-Lactate solution). Six weeks after the second injection, mice were challenged *i*.*c*. with 40-fold median lethal doses (LD_50_) of the CVS-11 strain. Body weight, clinical signs of disease and survival of infected mice were monitored daily. All RABV-G mRNA and HDC vaccinated mice were protected against lethal rabies virus challenge (**Fig 3A**). None of the RABV-G vaccine treated mice displayed any weight loss. Two mice treated with HDC showed a transient weight loss (10–20% of initial body weight), but survived the experiment (**Fig 3B**). In contrast, at day 9 after infection, first buffer treated mice had to be sacrificed due to pronounced weight loss (25% of initial body weight) and none in this group survived the infection. This challenge study clearly indicates that the RABV-G mRNA vaccine induces protective immunity against an otherwise lethal infection.

**Fig 4:**
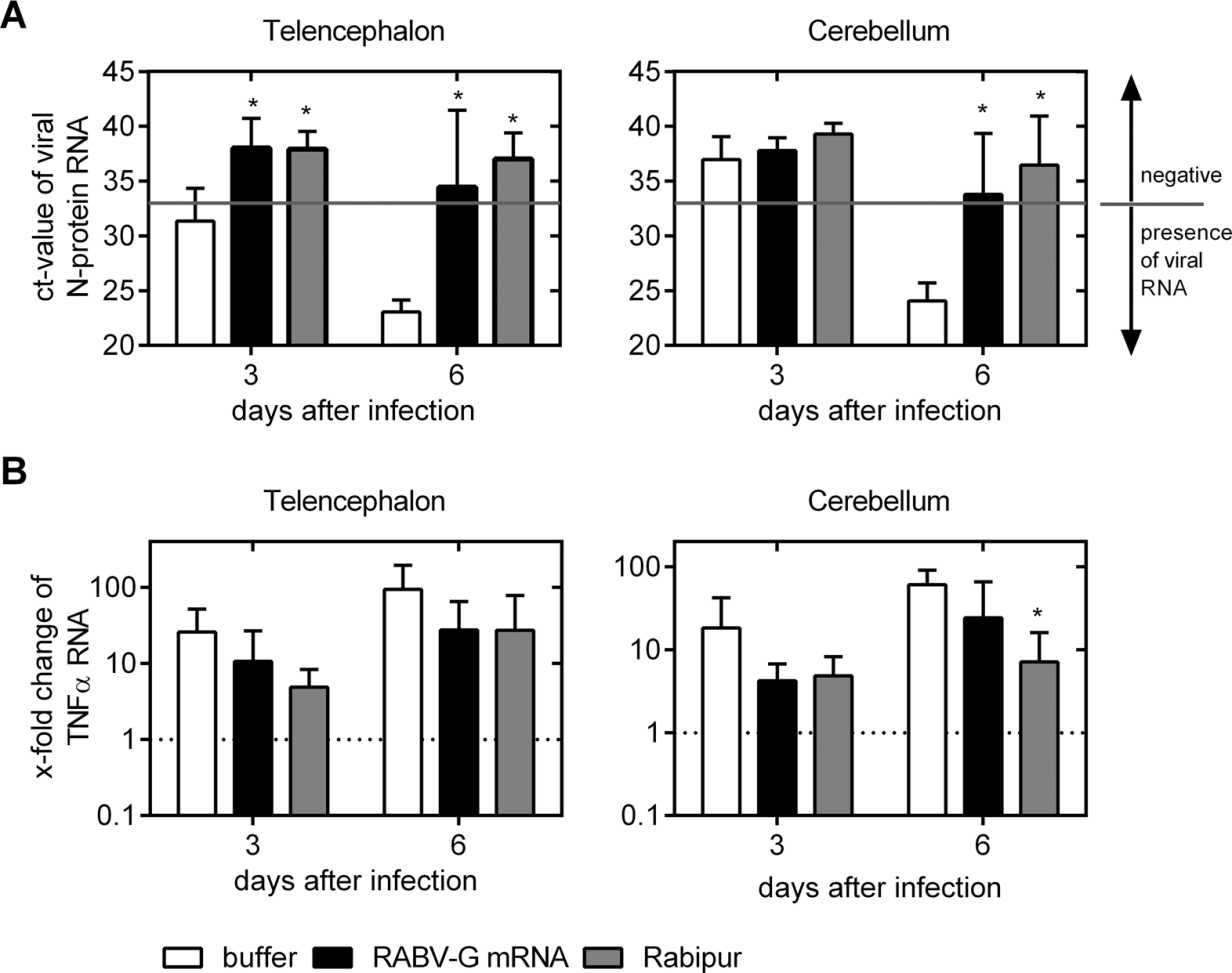
qRT-PCR analysis of viral N-protein encoding RNA and cellular TNFα mRNA transcript levels in brains 3 and 6 days after i.c. rabies challenge infection. Female BALB/c mice were injected with buffer alone (*white bar*), 80 μg RABV-G mRNA (*black bar*) or 0.1 human dose Rabipur (*grey bar*), on study days 0 and 21. 5 weeks after second immunization, mice were i.c. infected (into the telencephalon) with rabies virus. 3 and 6 days thereafter, mice were sacrificed. Perfused brains were divided into telencephalon *(left panel)* and cerebellum *(right panel)* and tested by qRT-PCR. ***(A)*** Detection of viral N-protein RNA. Ct-values are illustrated with a N-protein RNA detection limit of 33 cycles *(grey line)*. Ct-values below the detection limit are positive for virus RNA as indicated in the figure. Bars represent the mean and SD (n = 4/group). Significance between the buffer control group (*white bar*) and immunized groups was calculated using the one-way ANOVA Tukey’s multiple comparison test (* p<0.05). ***(B)*** TNFα mRNA levels in the telencephalon *(left panel)* and cerebellum *(right panel)*. RNA of brains from uninfected animals served as control. mRNA values of controls were defined as “1” (dotted line). Fold changes were calculated and bars represent the mean and SD (n = 4/group). Statistical significance to uninfected controls was tested with one-way ANOVA Tukey’s multiple comparison test with a significance level of 5% (*).

Having demonstrated protection against disease and death, we next analyzed cerebral viral loads in infected mice. As before, we injected BALB/c mice on days 0 and 21 with 80 μg RABV-G mRNA, 0.1 human doses of Rabipur or buffer. At day 56 of trial, all animals were infected with a 40-fold LD_50_ of CVS-11 by *i*.*c*. injection in the telencephalon. At days 3 and 6 after infection three animals per group were sacrificed. Perfused brains were tested for rabies virus replication by quantitative RT-PCR of RNA encoding the rabies nucleoprotein (N protein). At day 3 after infection a low quantity of N-protein mRNA was detectable in the telencephalon of animals treated with buffer (mean: 31.4 cycle threshold (ct)-value) whereas the cerebellum was negative for viral N-protein mRNA (mean above detection limit). Ct-values, however, decreased over the period of infection as at day 6 high viral loads were detected in both analyzed parts of the brains (mean ct-values: 23.1 (telencephalon) and 24.7 (cerebellum)). N-protein mRNA was not normalized to a cellular house-keeping gene as viral gene expression does not necessarily correlate with cellular gene expression. However, RNA concentration of each sample was determined and the amount of RNA used for the assay was adjusted accordingly. In contrast, all but one of the analyzed brains of mice immunized with RABV-G mRNA or Rabipur were negative for N-protein mRNA at both time points, demonstrating a strong suppression of viral replication below the detection limit after vaccination (**Fig 4A**). The brain of one mRNA vaccinated mouse was positive for viral N-protein RNA at day 6 after infection (telencephalon: ct-value of 25.4) indicating a non-protective response to the mRNA vaccine in one case.

The homeostasis of the CNS is maintained by the blood-brain-barrier (BBB), a complex system of different cell types [41]. It has been reported that rabies virus-induced encephalitis results in an elevation of different pro-inflammatory cytokines. Increased levels of TNFα and IFN-γ have been causally linked to increased BBB permeability which also allows the strong influx of immune factors [42,43]. We analyzed mRNA levels of TNFα in brains at 3 and 6 days after infection by qRT-PCR together with the viral N protein analyzed above. The mRNA levels of TNFα in the telencephalon rose rapidly in the buffer injected group up to 25.8-fold at day 3 and 94.6-fold at day 6 after infection compared to non-infected controls, which reflects the early activation of this pro-inflammatory cytokine as response to the virus infection. Also in the cerebellum, the level of TNFα mRNA transcripts rose up to 60.5-fold at day 6 compared to uninfected controls, indicating a strong pro-inflammatory innate immune response upon viral spread. In mRNA and Rabipur vaccinated mice TNFα mRNA transcript level was reduced compared to mock vaccinated control group at both analysis time points and different brain sections (**Fig 4B**).

### Importance of CD4+ T cells for the induction of a protective immune response

**Fig 5:**
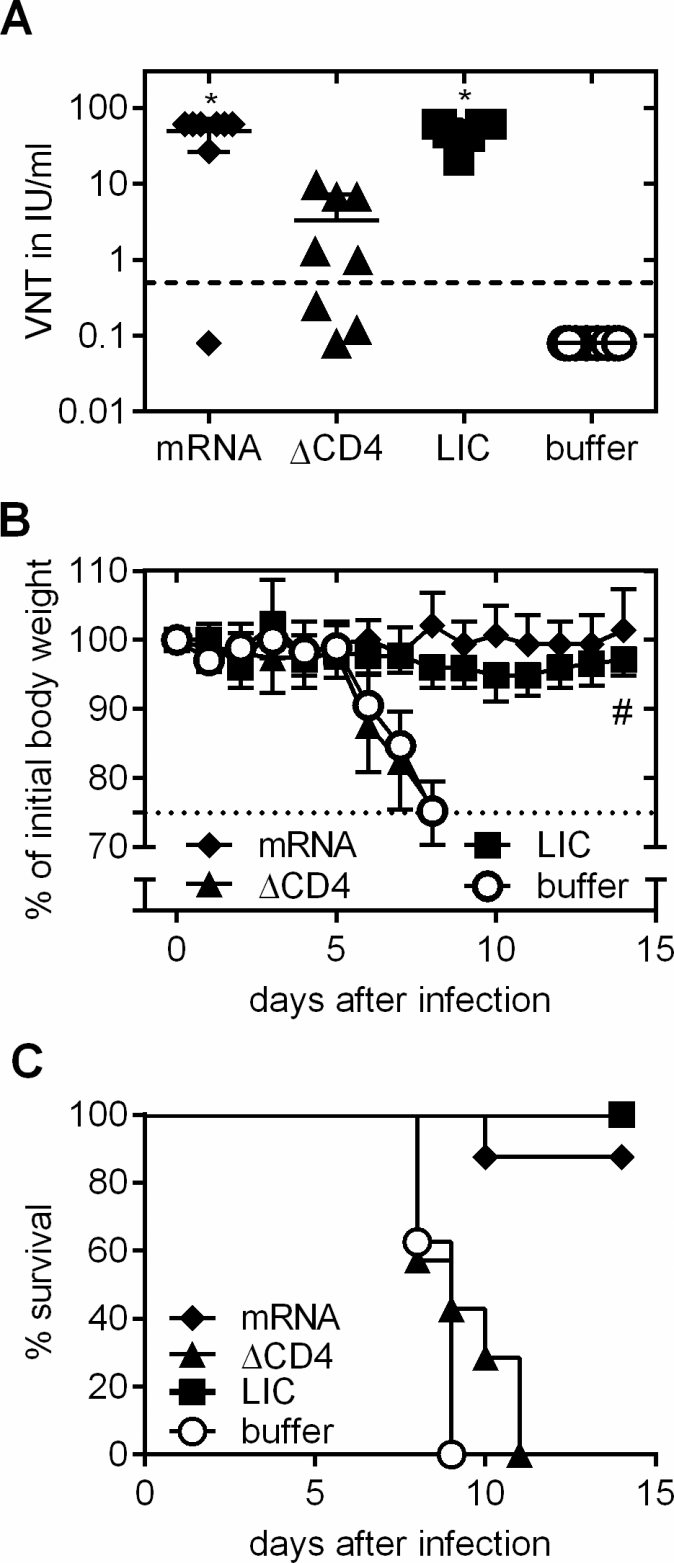
CD4+ T cell depletion during immunization phase followed by challenge infection. Female BALB/c mice were vaccinated with RABV-G mRNA, Rabipur (LIC) or injection buffer alone on days 0 and 21. The CD4+ T cell population was specifically depleted in one mRNA-receiving group by injection of anti-CD4 antibody on days -1, 1, 20 and 22. ***(A)*** Evaluation of rabies virus neutralizing antibodies in sera of control, vaccinated or vaccinated and CD4-depleted mice 4 weeks after the second immunization using the FAVN test (n = 8/group). Mean and SD is presented. Significance between the buffer control group and immunized groups was calculated using the one-way ANOVA Tukey’s multiple comparison test (* p<0.05). ***(B*, *C)*** Mice were challenged with a lethal dose of rabies virus and ***(B)*** body weight was monitored daily. A loss of 25% of initial body weight was defined as humane endpoint. Mean and SD is presented. ***(C)*** Survival of challenged mice. ^**#**^ note: the sacrificed mouse in the RABV-G mRNA (black diamond) group was excluded from the body weight analysis to demonstrate the healthy state of the remaining animals. This mouse was also negative for rabies specific antibodies (see Fig 5A).

The RABV-G mRNA vaccine induced strong CD4+ T cell responses compared to licensed rabies vaccine (**Fig 2B and 2D**). In order to assess the mode of action of the mRNA based rabies vaccine, we addressed the role of mRNA vaccine-induced CD4+ T cells in forming VN antibodies and in protecting against challenge infection. To this end, in mRNA vaccinated BALB/c mice CD4+ T cells were depleted during both, prime and boost immunization periods (around days 0 and 21) by injecting a specific anti-CD4 monoclonal antibody [31]. Analysis by flow cytometry confirmed a drastic reduction of the CD4+ T cell population by about 98.6% at day 6 and 97.1% at day 23 after first immunization (**S4 Fig**). 28 days after the second immunization, VN antibodies in sera were quantified (**Fig 5A**). Mice with intact CD4+ cell compartment immunized with either RABV-G mRNA or Rabipur had comparable VN titers with means of about 50 IU/ml (undepleted positive controls). In the CD4-depleted, mRNA immunized group VN titers were strongly reduced: 3 out of 8 mice showed VN titers below 0.5 IU/ml and the mean titer of the groups was about 3 IU/ml, i.e. more than 15-fold reduced compared to the CD4-intact, mRNA-vaccinated group. The functional role of CD4+ T cells for the generation of neutralizing antibodies or direct antiviral protection was further reflected by survival rates of mice after lethal *i*.*c*. challenge with CVS-11: All mice vaccinated with Rabipur (undepleted positive control) survived the challenge infection while buffer injected mice (negative controls) had to be sacrificed by day nine post challenge due to dramatic weight loss (**Fig 5C**). In groups with an intact CD4+ T cell compartment during prophylactic immunization, more than 87% of mRNA-vaccinated mice survived infection (one out of eight mRNA-immunized mice without detectable virus neutralizing antibodies after immunization had to be sacrificed on day ten due to pronounced weight loss) (**Fig 5B**). In the CD4-depleted, mRNA vaccinated group, all mice had to be sacrificed within 11 days after infection with similar kinetics as in the negative control group (**Fig 5C**) in line with the strongly reduced VN titers in this group.

### Seroconversion in newborn and adult pigs

We next analyzed immunogenicity in domestic pigs, a large animal model that exhibits relevant physiological and anatomical similarities to humans [44,45].

**Fig 6:**
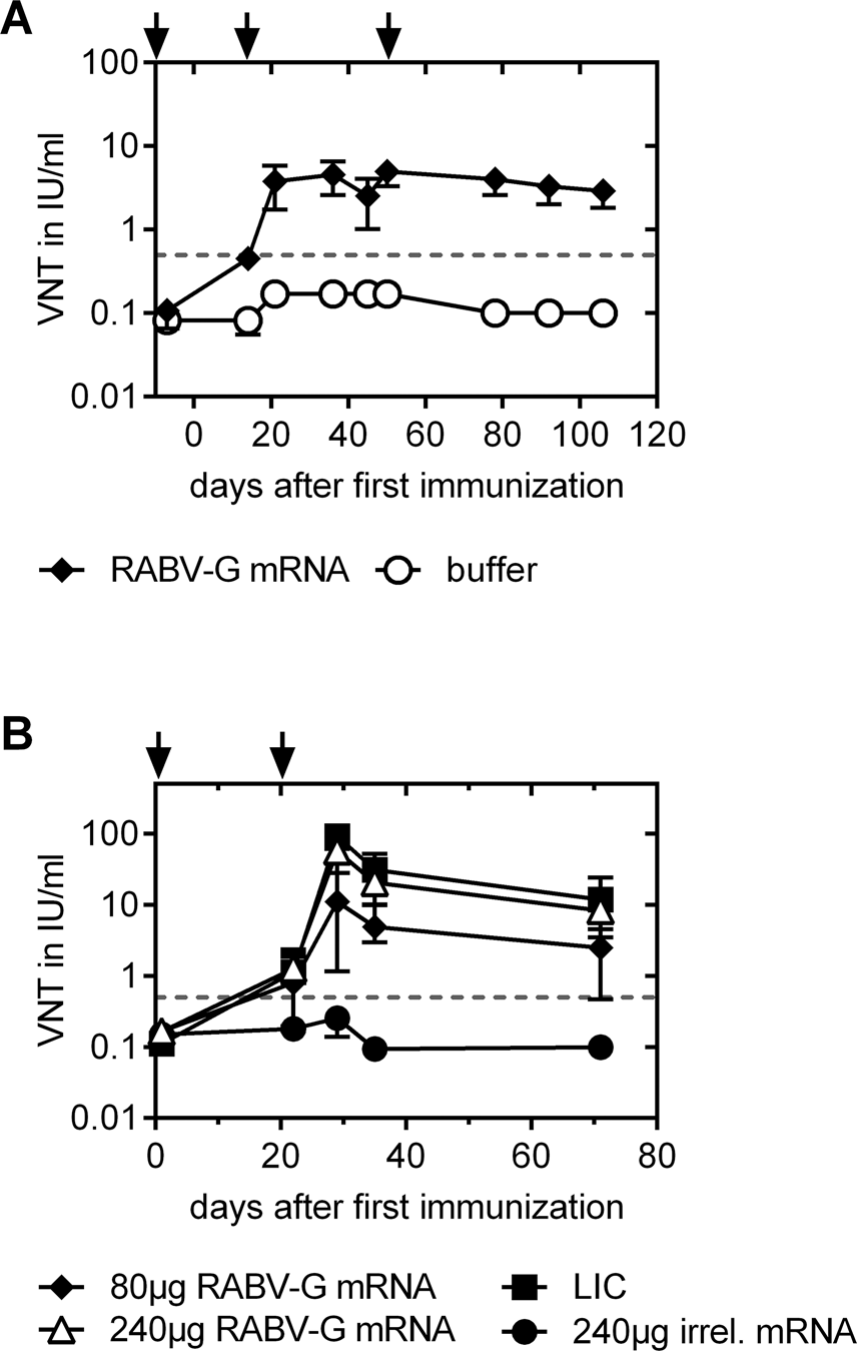
Seroconversion profile after RABV-G mRNA and Rabipur (LIC) vaccination in domestic pigs. Immunization points are indicated by arrows. ***(A)*** Adult pigs received intradermal immunizations on days 0, 14, 49 with either 80 μg RABV-G mRNA or injection buffer alone. At different time points after immunization, sera were tested for rabies-specific neutralizing antibodies using FAVN testing (n = 6 pigs/group). ***(B)*** Newborn piglets were immunized in their first week of life and 3 weeks later with 80 μg or 240 μg RABV-G mRNA (i.d.) or a full human dose of Rabipur (LIC; i.m.). As a negative control, animals were immunized with 240 μg of an irrelevant mRNA. At different time points blood samples were taken and sera were tested for virus-neutralizing antibodies using the FAVN test (n = 5 or n = 6 (240 μg) piglets/group). In both graphs mean and SD is illustrated.

First, we investigated whether the RABV-G mRNA vaccine induces protective antibody titers in large animals with a body weight above 20 kg. Therefore, adult pigs at 6–8 weeks of age (22.2–31.2 kg body weight) were vaccinated intradermally with 80 μg RABV-G mRNA or buffer at days 0, 14 and 49 (**Fig 6A**). mRNA was applied at a three dose schedule as recommended for licensed vaccine benchmarks, however, longer intervals were chosen between vaccinations to assess the impact of each single prime and follow-up booster vaccination. The first two vaccinations with RABV-G mRNA led to seroconversion of all animals with antibody titers >0.5 IU/ml. The third vaccination at day 49 did not further increase RABV-G specific neutralizing antibody levels. Eight weeks post second boost RABV-G specific neutralizing antibodies had a mean titer of 2.9 IU/ml and were still above the 0.5 IU/ml limit. Furthermore, we assessed whether the RABV-G mRNA vaccine induced protective antibody titers in newborn piglets with an immature immune system, thereby extending former findings on vaccine immunogenicity in newborn mice [16]. Piglets (approx. 1.5 kg body weight) were immunized intradermally with 80 μg or 240 μg RABV-G mRNA vaccine doses within three days after birth and corresponding booster injections three weeks later. One group received 240 μg of irrelevant mRNA. For comparison with a licensed vaccine, one group of piglets received a full human dose of Rabipur that was injected intramuscularly as recommended. All specifically immunized piglets rapidly seroconverted to neutralizing antibody titers >0.5 IU/ml which was already reached at day 28, the first analysis time point after the initial vaccination. Responses to RABV-G mRNA vaccine were dose dependent. Piglets receiving 80 μg RABV-G mRNA developed mean protective titers of 11.0 IU/ml at day 28 and 2.5 IU/ml at day 70, while piglets immunized with the 240 μg dose revealed higher mean antibody titers of 56.8 IU/ml at day 28 and 8.3 IU/ml at day 70 that were comparable to those induced by full doses of the licensed human vaccine (**Fig 6B**). These data demonstrate that RABV-G vaccine is immunogenic in newborn piglets, leading to rapid seroconversion and indicating comparable VN titer kinetics for mRNA vaccine compared to the licensed Rabipur vaccine.

## Discussion

Rabies is a severe neurological disease with a mortality rate of almost 100% in symptomatic individuals for whom no effective therapy exists [46]. The disease remains endemic in developing countries where stray dogs transmitting the disease are not controlled and the imperative need for better vaccines is illustrated by the fact that in Asia and Africa 3–4 billion people are still considered at risk of exposure to rabies virus [47]. Though vaccination of dogs combined with human post-exposure prophylaxis programs became a focus in rabies elimination, a post-exposure prophylaxis especially in countries at high risk face different difficulties, including delays in patient access to suitable medical care [48]. Efficacy, availability and affordable cost for prophylactic vaccines for human use may significantly contribute to resolve this problem, since the amount of vaccinations in this setting is reduced to a single vaccination and rabies immune globulin is dispensable. Messenger RNA is a promising new vector format that may allow for significant improvements in vaccine manufacture and supply [17,18].

In the present study, we demonstrated the immunogenicity and protective efficacy of an experimental mRNA-based rabies vaccine in animals. This mRNA vaccine encoding the Rabies virus glycoprotein was immunogenic upon two injections at microgram doses and resulted in the induction of virus neutralizing antibodies, antigen-specific CD4+ T cells and CD8+ T cells (Fig 1). Protection against infectious challenge was stringently demonstrated in mice. As the neurotropic virus was directly injected into the central nervous system (CNS), the time frame for effective neutralization of infectious particles by pre-existing antibodies is extremely short. Using qRT-PCR based analysis of the viral nucleoprotein gene it was revealed that spread of the virus was strongly inhibited by RABV-G mRNA vaccination. Protection against intracerebral infection may result from antibodies that neutralize free viral particles before entering nerve cells [49,50] or from invading B cells that directly produce neutralizing antibodies in the CNS [51]. We found a clear correlation between the induction of protective virus neutralization titers and survival upon infectious challenge. Our finding that depletion of CD4+ T cells during the immunization phase results in a loss of protection against lethal challenge infection further indicates the need for T cell help in mounting a protective neutralizing antibody response. Interestingly, some mice in the T cell depleted group showed VN titers above 0.5 IU/ml but succumbed to the challenge infection. Similar observations have been reported by others as well [52,53] and, moreover, a study analyzing the correlation between VN titer and protection against i.c. challenge for BALB/c mice in the NIH potency test for rabies vaccines reported that individual animals with titer up to 7.8 IU/ml were not protected [49]. This might indicate that the titer of 0.5 IU/ml is rather a surrogate marker for protection for a natural rabies infection (e.g. intramuscular in the periphery) than for the laboratory intracerebral infection in our experimental setting.

Concerning the induction of neutralizing antibodies, this finding indicates a comparable mode of action for the mRNA vaccine format as seen for cell based vaccines and other vaccine formats [3,13].

We analyzed TNFα expression in brains of vaccinated and control animals after rabies infection. In the brains of RABV-G mRNA or Rabipur vaccinated mice, the TNFα mRNA expression rose but to lower levels compared to buffer immunized animals. Though not statistically significant, the data indicate a reduced innate inflammatory reaction which might result in the maintenance of brain homeostasis upon vaccination with RABV-G mRNA or licensed rabies vaccine. TNFα appears to be an important cytokine in the brain for viral clearance with the function to attract effector cells, and therefore also in immunized mice the elevation of TNFα mRNA expression was expected for initiating viral clearance by effector cells. However, while TNFα plays a major role in attracting immune cells and thereby boosting inflammation that may lead to rabies-encephalitis, the direct impact of TNFα on BBB integrity remains controversial [43,54]. In buffer-treated mice we found viral replication in the telencephalon (the site of injection) and spreading to the cerebellum. In addition, both telencephalon and cerebellum of these animals showed increased TNFα expression consistent with the idea of a compromised BBB integrity and inflammation processes throughout the whole brain. In contrast and disregarding a single non-protected mouse, RABV-G mRNA or Rabipur vaccinated mice survived challenge infections without clear signs of viral replication and spread into the cerebellum in quantitative RT-PCR analyses as early as 3 days after infection. These findings underline the robust protection by the mRNA vaccine.

Immunogenicity of RABV-G mRNA vaccine was further assessed in domestic pigs. In adult pigs, titers reached the protective limit of ≥0.5 IU/ml after the first immunization and could be further increased by booster vaccination, then remaining stable throughout the experiment. Interestingly, a third vaccination did not result in a further strong titer increase, suggesting a maximal humoral immune response by the prime-boost strategy in pigs. Furthermore, immunogenicity of the RABV-G mRNA was also investigated in newborn piglets, again showing neutralizing antibody titers after the first vaccination in the protective range that were further increased after the booster vaccination. The kinetic of virus neutralizing antibody response for the RABV-G mRNA was comparable to the full human dose of the licensed rabies vaccine Rabipur. These data support and extend previous findings for mRNA vaccines against influenza in adult and newborn mice [16].

A clinical phase I study to test safety and immunogenicity of this RABV-G mRNA vaccine in healthy volunteers has been initiated (EudraCT no. 2013-002171-17, NCT02241135). In summary, we further substantiated and extended previously published work on non-replicating influenza-specific mRNA vaccines [16], proving the protective efficacy of mRNA vaccination in a second, harsh viral challenge model. Unlike in the case of influenza, pre-existing immunity was not expected to affect experimental outcomes especially in pigs. This allowed for *bona fide* assessments of vaccine immunogenicity in naïve individuals on the basis of a widely recognized and standardized functional correlate of protection (virus neutralizing titers). These findings, together with the stability of mRNA vaccine [16,55] and projected as well as observed cost for production of mRNA vaccines [19–21] further substantiate the assumption that this new vaccination strategy may be extended to the prophylaxis and treatment of infectious diseases in general.

## Supporting Information

S1 Fig Nucleotide sequences of mRNAs used for vaccinations. RABV-G A and B contain the identical open reading frame, encoding the RABV-G protein. The two RNA constructs differ in the non-coding 3’ and 5’ untranslated regions (UTRs).
(DOCX)

S2 Fig Expression of RABV-G mRNA vaccine in HeLa cells. Cells were transfected with 5 μg of RABV-G mRNA or 5 μg HA mRNA encoding the hemagglutinin (HA) of influenza virus A/Netherlands/602/2009. 24 h after transfection, cells were stained with a monoclonal mouse anti-rabies antibody and a FITC labelled goat anti-mouse IgG. Expression was detected by flow cytometric analysis of FITC positive cells. For control unstained, RABV-G mRNA transfected cells were analyzed.
(DOCX)

S3 Fig Induction of RABV-G specific CD4+ T cell is dose dependent. Female BALB/c mice were vaccinated twice (days 0 and 21) with 80, 20, 10, 5, and 1 μg RABV-G mRNA, 0.1 human dose Rabipur (LIC) or buffer. Ten weeks after boost, animals were sacrificed and IFN-γ and TNFα double positive antigen-specific CD4+ T cells were analysed by flow cytometry (n = 8/group). Mean and SD (n = 8/group) is presented.
(DOCX)

S4 Fig Depletion CD4+ T cell in mice during immunization phase. Mice were immunized at day 0 and 21. One day before and one day after immunization, CD4+ T cells were depleted using a monoclonal anti-CD4 antibody. Depletion was controlled by flow cytometry. ***(A)*** Flow cytometry analysis of single probes 2 and 23 days after first immunization as well as of untreated controls indicating the percental fraction of CD3+CD4+ cells within the gated cells. ***(B)*** Summary of all evaluated data (n = 4/group). Statistical significance was tested with one-way ANOVA test using Dunnett’s multiple comparisons test compared to control (* p<0.0001).
(DOCX)
