# Supplementary material for: An mRNA Vaccine Encoding Rabies Virus Glycoprotein Induces Protection against Lethal Infection in Mice and Correlates of Protection in Adult and Newborn Pigs
Source: PLoS Negl Trop Dis. 2016 Jun 23;10(6):e0004746. doi: 10.1371/journal.pntd.0004746 (PMC4918980; doi:10.1371/journal.pntd.0004746)
Supplement: S1 Fig — RABV-G A and B contain the identical open reading frame, encoding the RABV-G protein. The two RNA constructs differ in the non-coding 3’ and 5’ untranslated regions (UTRs). (DOCX) [file pntd.0004746.s001.docx]

**Figure S1: Nucleotide sequences of mRNAs used for vaccinations**

The two sequences of the RABV-G, A and B, contain the identical open reading frame, encoding the RABV-G protein of the Pasteur strain (GenBank accession number: AAA47218.1). The two RNA constructs differ in the non-coding 3’ and 5’ untranslated regions (UTRs).

**RABV-G mRNA A**

GGGAGAAAGCUUACCAUGGUGCCCCAGGCCCUGCUCUUCGUCCCGCUGCUGGUGUUCCCCCUCUGCUUCGGCAAGUUCCCCAUCUACACCAUCCCCGACAAGCUGGGGCCGUGGAGCCCCAUCGACAUCCACCACCUGUCCUGCCCCAACAACCUCGUGGUCGAGGACGAGGGCUGCACCAACCUGAGCGGGUUCUCCUACAUGGAGCUGAAGGUGGGCUACAUCAGCGCCAUCAAGAUGAACGGGUUCACGUGCACCGGCGUGGUCACCGAGGCGGAGACCUACACGAACUUCGUGGGCUACGUGACCACCACCUUCAAGCGGAAGCACUUCCGCCCCACGCCGGACGCCUGCCGGGCCGCCUACAACUGGAAGAUGGCCGGGGACCCCCGCUACGAGGAGUCCCUCCACAACCCCUACCCCGACUACCACUGGCUGCGGACCGUCAAGACCACCAAGGAGAGCCUGGUGAUCAUCUCCCCGAGCGUGGCGGACCUCGACCCCUACGACCGCUCCCUGCACAGCCGGGUCUUCCCCGGCGGGAACUGCUCCGGCGUGGCCGUGAGCUCCACGUACUGCAGCACCAACCACGACUACACCAUCUGGAUGCCCGAGAACCCGCGCCUGGGGAUGUCCUGCGACAUCUUCACCAACAGCCGGGGCAAGCGCGCCUCCAAGGGCAGCGAGACGUGCGGGUUCGUCGACGAGCGGGGCCUCUACAAGUCCCUGAAGGGGGCCUGCAAGCUGAAGCUCUGCGGCGUGCUGGGCCUGCGCCUCAUGGACGGGACCUGGGUGGCGAUGCAGACCAGCAACGAGACCAAGUGGUGCCCCCCCGGCCAGCUGGUCAACCUGCACGACUUCCGGAGCGACGAGAUCGAGCACCUCGUGGUGGAGGAGCUGGUCAAGAAGCGCGAGGAGUGCCUGGACGCCCUCGAGUCCAUCAUGACGACCAAGAGCGUGUCCUUCCGGCGCCUGAGCCACCUGCGGAAGCUCGUGCCCGGGUUCGGCAAGGCCUACACCAUCUUCAACAAGACCCUGAUGGAGGCCGACGCCCACUACAAGUCCGUCCGCACGUGGAACGAGAUCAUCCCGAGCAAGGGGUGCCUGCGGGUGGGCGGCCGCUGCCACCCCCACGUCAACGGGGUGUUCUUCAACGGCAUCAUCCUCGGGCCCGACGGCAACGUGCUGAUCCCCGAGAUGCAGUCCAGCCUGCUCCAGCAGCACAUGGAGCUGCUGGUCUCCAGCGUGAUCCCGCUCAUGCACCCCCUGGCGGACCCCUCCACCGUGUUCAAGAACGGGGACGAGGCCGAGGACUUCGUCGAGGUGCACCUGCCCGACGUGCACGAGCGGAUCAGCGGCGUCGACCUCGGCCUGCCGAACUGGGGGAAGUACGUGCUGCUCUCCGCCGGCGCCCUGACCGCCCUGAUGCUGAUCAUCUUCCUCAUGACCUGCUGGCGCCGGGUGAACCGGAGCGAGCCCACGCAGCACAACCUGCGCGGGACCGGCCGGGAGGUCUCCGUGACCCCGCAGAGCGGGAAGAUCAUCUCCAGCUGGGAGUCCUACAAGAGCGGCGGCGAGACCGGGCUGUGAGGACUAGUUAUAAGACUGACUAGCCCGAUGGGCCUCCCAACGGGCCCUCCUCCCCUCCUUGCACCGAGAUUAAUAAAAAAAAAAAAAAAAAAAAAAAAAAAAAAAAAAAAAAAAAAAAAAAAAAAAAAAAAAAAAAAAUGCAUCCCCCCCCCCCCCCCCCCCCCCCCCCCCCCCAAAGGCUCUUUUCAGAGCCACCAGAAUU

**RABV-G mRNA B**

GGGGCGCUGCCUACGGAGGUGGCAGCCAUCUCCUUCUCGGCAUCAAGCUUACCAUGGUGCCCCAGGCCCUGCUCUUCGUCCCGCUGCUGGUGUUCCCCCUCUGCUUCGGCAAGUUCCCCAUCUACACCAUCCCCGACAAGCUGGGGCCGUGGAGCCCCAUCGACAUCCACCACCUGUCCUGCCCCAACAACCUCGUGGUCGAGGACGAGGGCUGCACCAACCUGAGCGGGUUCUCCUACAUGGAGCUGAAGGUGGGCUACAUCAGCGCCAUCAAGAUGAACGGGUUCACGUGCACCGGCGUGGUCACCGAGGCGGAGACCUACACGAACUUCGUGGGCUACGUGACCACCACCUUCAAGCGGAAGCACUUCCGCCCCACGCCGGACGCCUGCCGGGCCGCCUACAACUGGAAGAUGGCCGGGGACCCCCGCUACGAGGAGUCCCUCCACAACCCCUACCCCGACUACCACUGGCUGCGGACCGUCAAGACCACCAAGGAGAGCCUGGUGAUCAUCUCCCCGAGCGUGGCGGACCUCGACCCCUACGACCGCUCCCUGCACAGCCGGGUCUUCCCCGGCGGGAACUGCUCCGGCGUGGCCGUGAGCUCCACGUACUGCAGCACCAACCACGACUACACCAUCUGGAUGCCCGAGAACCCGCGCCUGGGGAUGUCCUGCGACAUCUUCACCAACAGCCGGGGCAAGCGCGCCUCCAAGGGCAGCGAGACGUGCGGGUUCGUCGACGAGCGGGGCCUCUACAAGUCCCUGAAGGGGGCCUGCAAGCUGAAGCUCUGCGGCGUGCUGGGCCUGCGCCUCAUGGACGGGACCUGGGUGGCGAUGCAGACCAGCAACGAGACCAAGUGGUGCCCCCCCGGCCAGCUGGUCAACCUGCACGACUUCCGGAGCGACGAGAUCGAGCACCUCGUGGUGGAGGAGCUGGUCAAGAAGCGCGAGGAGUGCCUGGACGCCCUCGAGUCCAUCAUGACGACCAAGAGCGUGUCCUUCCGGCGCCUGAGCCACCUGCGGAAGCUCGUGCCCGGGUUCGGCAAGGCCUACACCAUCUUCAACAAGACCCUGAUGGAGGCCGACGCCCACUACAAGUCCGUCCGCACGUGGAACGAGAUCAUCCCGAGCAAGGGGUGCCUGCGGGUGGGCGGCCGCUGCCACCCCCACGUCAACGGGGUGUUCUUCAACGGCAUCAUCCUCGGGCCCGACGGCAACGUGCUGAUCCCCGAGAUGCAGUCCAGCCUGCUCCAGCAGCACAUGGAGCUGCUGGUCUCCAGCGUGAUCCCGCUCAUGCACCCCCUGGCGGACCCCUCCACCGUGUUCAAGAACGGGGACGAGGCCGAGGACUUCGUCGAGGUGCACCUGCCCGACGUGCACGAGCGGAUCAGCGGCGUCGACCUCGGCCUGCCGAACUGGGGGAAGUACGUGCUGCUCUCCGCCGGCGCCCUGACCGCCCUGAUGCUGAUCAUCUUCCUCAUGACCUGCUGGCGCCGGGUGAACCGGAGCGAGCCCACGCAGCACAACCUGCGCGGGACCGGCCGGGAGGUCUCCGUGACCCCGCAGAGCGGGAAGAUCAUCUCCAGCUGGGAGUCCUACAAGAGCGGCGGCGAGACCGGGCUGUGAGGACUAGUGCAUCACAUUUAAAAGCAUCUCAGCCUACCAUGAGAAUAAGAGAAAGAAAAUGAAGAUCAAUAGCUUAUUCAUCUCUUUUUCUUUUUCGUUGGUGUAAAGCCAACACCCUGUCUAAAAAACAUAAAUUUCUUUAAUCAUUUUGCCUCUUUUCUCUGUGCUUCAAUUAAUAAAAAAUGGAAAGAACCUAGAUCUAAAAAAAAAAAAAAAAAAAAAAAAAAAAAAAAAAAAAAAAAAAAAAAAAAAAAAAAAAAAAAAAUGCAUCCCCCCCCCCCCCCCCCCCCCCCCCCCCCCCAAAGGCUCUUUUCAGAGCCACCAGAAUU
