# Supplementary figures and images for: An mRNA Vaccine Encoding Rabies Virus Glycoprotein Induces Protection against Lethal Infection in Mice and Correlates of Protection in Adult and Newborn Pigs
Source: PLoS Negl Trop Dis. 2016 Jun 23;10(6):e0004746. doi: 10.1371/journal.pntd.0004746 (PMC4918980; doi:10.1371/journal.pntd.0004746)

**Figure S2: Expression of RABV-G mRNA vaccine in HeLa cells.**


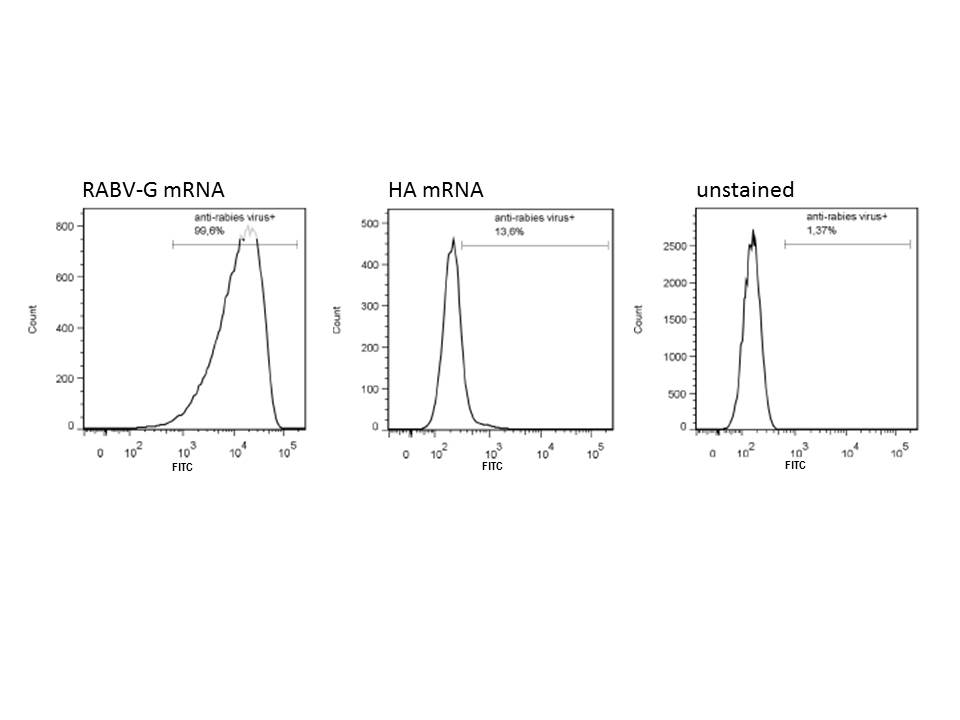

Supplement: S2 Fig — Cells were transfected with 5 μg of RABV-G mRNA or 5 μg HA mRNA encoding the hemagglutinin (HA) of influenza virus A/Netherlands/602/2009. 24 h after transfection, cells were stained with a monoclonal mouse anti-rabies antibody and a FITC labelled goat anti-mouse IgG. Expression was detected by flow cytometric analysis of FITC positive cells. For control unstained, RABV-G mRNA transfected cells were analyzed. (DOCX) [file pntd.0004746.s002.docx]

**
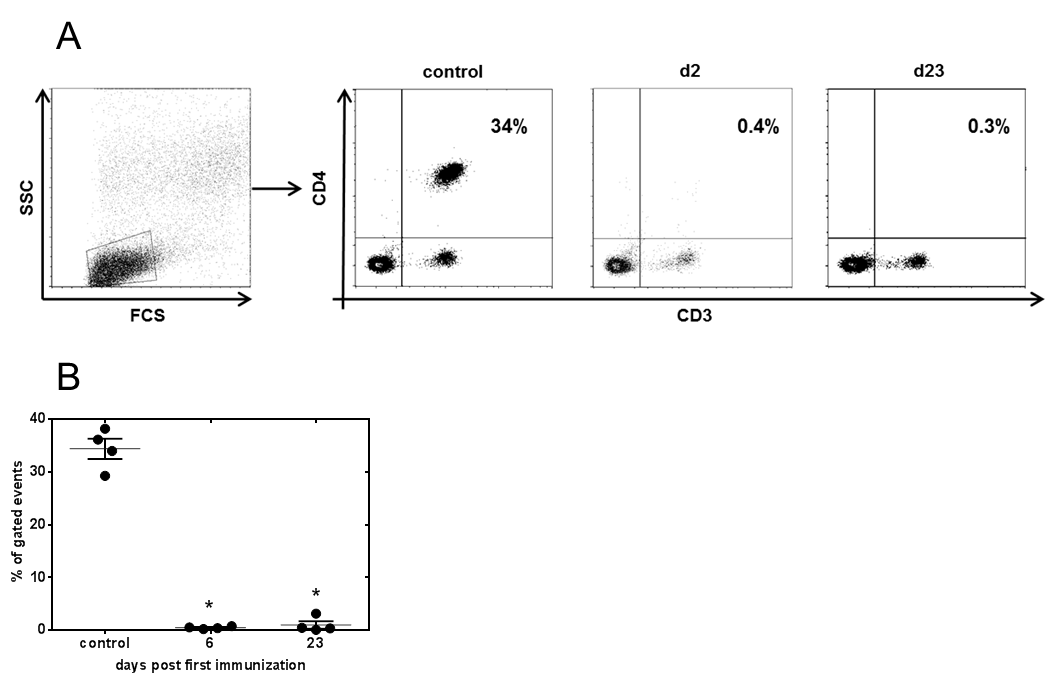
Figure S3: induction of antigen specific CD4 T cells is dose dependent**

Supplement: S3 Fig — Female BALB/c mice were vaccinated twice (days 0 and 21) with 80, 20, 10, 5, and 1 μg RABV-G mRNA, 0.1 human dose Rabipur (LIC) or buffer. Ten weeks after boost, animals were sacrificed and IFN-γ and TNFα double positive antigen-specific CD4+ T cells were analysed by flow cytometry (n = 8/group). Mean and SD (n = 8/group) is presented. (DOCX) [file pntd.0004746.s003.docx]

**
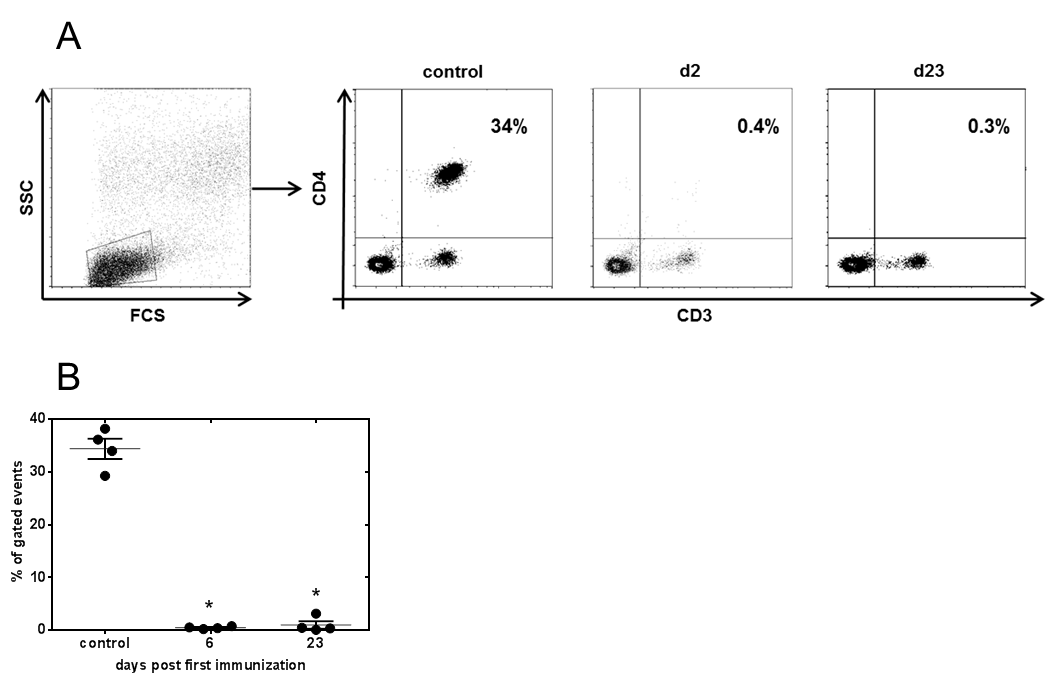
Figure S4: Depletion CD4+ T cell in mice during immunization phase.**

Supplement: S4 Fig — Mice were immunized at day 0 and 21. One day before and one day after immunization, CD4+ T cells were depleted using a monoclonal anti-CD4 antibody. Depletion was controlled by flow cytometry. (A) Flow cytometry analysis of single probes 2 and 23 days after first immunization as well as of untreated controls indicating the percental fraction of CD3+CD4+ cells within the gated cells. (B) Summary of all evaluated data (n = 4/group). Statistical significance was tested with one-way ANOVA test using Dunnett’s multiple comparisons test compared to control (* p<0.0001). (DOCX) [file pntd.0004746.s004.docx]
